# The level of knowledge about palliative care in Iranian patients with cancer

**DOI:** 10.1186/s12904-022-00920-9

**Published:** 2022-03-10

**Authors:** Dadgari Atena, Bagheri Imane, Rassouli Maryam, Salmani Naiire, Tahani Fatemeh

**Affiliations:** 1 Nursing Faculty, Meybod Nursing School, Shahid Sadoughi University of Medical Sciences, Yazd, Iran; 2 Ph.D Nursing Student, College of Nursing and Midwifery, Isfahan University of Medical Sciences, Isfahan, Iran; 3 PhD. RN Cancer Research Center, Shahid Beheshti University of Medical Sciences, Tehran, Iran; 4 Hematology and Oncology Research Center, Shahid Sadoughi University of Medical Sciences, Yazd, Iran; 5 Yazd, Iran; 6 Oncology Nurse, Shahid Sadoughi Hospital, Shahid Sadoughi University of Medical Sciences, Yazd, Iran

**Keywords:** Cancer, Knowledge, Palliative Care, Patients

## Abstract

**Background:**

Patient awareness plays an important role in integrating palliative care into the patient care process. Lack of awareness can create a negative attitude towards palliative care and affect patients' decisions during the treatment process. The present study was conducted to determine the level of knowledge about palliative care in Iranian patients with cancer.

**Methods:**

The descriptive study was conducted with a convenience sample of 103 cancer patients admitted to an oncology center in Yazd, Iran, A three-part questionnaire including demographic information, sources of palliative care information and the Palliative Care Knowledge Scale (PaCKS) was used to collect data. All statistical analyses were performed using software SPSS 21.

**Results:**

The mean age of patients was 36.2 ± 13.5 years. Of the total, 38.8% of patients received information about palliative care through the media and 36.9% from the treatment team. On a scale of 0 to 13, the mean PaCKS score was 6.7 ± 3.7. A ‘good’ level of knowledge was reported by 29.1% of participants; however 84.5% stated that they should leave other doctors at the time of receiving palliative care, 71.8% considered palliative care for patients in the last six months of life, 84.5% considered palliative care for patients with cancer, and 70.9% stated that palliative care encourages people to discontinue treatments aimed at treating their disease.

**Conclusion:**

Our study found most cancer patients have a moderate to weak level of knowledge and considerable misinformation about palliative care, which highlights the importance of providing palliative care education. The development of training programs in this area could play an effective role in improving patients' knowledge of palliative care.

## Background

Cancer is one of the most challenging diseases worldwide of the current century [1]. After cardiovascular diseases and accidents, cancer is the third leading cause of death in Iran [2]; by 2025, there are expected to be more than 130,000 new cases of cancer in Iran [3]. This disease is a potential threat to life and can have adverse effects on the physical, psychological, social, economic and overall quality of life of patients, with 60–90% of patients having physical symptoms and complications such as fatigue, nausea, pain, diarrhea, from loss of appetite and 40% experiencing symptoms such as anxiety and depression [4, 5].

Palliative care is an interdisciplinary approach; the World Health Organization has introduced palliative care as a way to improve the quality of life of patients with incurable diseases and their families [6]. Palliative care begins with the diagnosis of the disease and continues throughout the illness [7]. and focuses on prevention or relief of the patient's suffering through early identification, evaluation, and treatment of pain and other problems of physical, psychological and social dimensions [8].

The integration of palliative care in the care program of cancer patients is an essential component for care management [9]. Although attention to palliative care is increasing today [10], many patients who can benefit from this type of care either never go into palliative care or receive it late [11]. In the United States the median time between palliative care referral and death was 72.5 days, with a mean 126.5 days [12].

While patients who have early referral to palliative care centers experience improved physical symptoms (dyspnea and fatigue) and anxiety, increase in quality of life [10, 13], survival time [10], tendency for death to occur at home [9], and satisfaction with care [13], and reduction in depression [10], visits for immediate care [14], Emergency visits, and hospital admissions [9]. In contrast, patients who have not received palliative care at all or who are late to this type of care are more likely to receive invasive treatments, including chemotherapy, and their risk of death from treatment complications increases [15].

Often, palliative care is delayed until patients are unresponsive to other treatments, even though integrating this type of care during different stages of treatment can reduce the severity of symptoms. The initiation of palliative care at the time of diagnosis provides a supportive partnership for the treatment team and as the disease progresses, the need for palliative care increases [16].

Therefore, it is recommended that palliative care be started in the first 8 weeks after the onset of the disease [17] and the American Society of Oncology Guidelines recommend that palliative care be introduced and started at the onset of cancer for some patients [18]. In order to integrate palliative care more quickly into the process of caring for cancer patients, it is essential to identify barriers that prevent patients from being referred to palliative care centers; among the various barriers, failure to meet patients' information needs has been identified as a major barrier. [19]. Beerneart et al. stated 30% of patients with a predicted lifespan of less than 6 months had limited information about palliative care [20]. Further, Zafar et al. reported that only 25% of patients with advanced cancer received information about palliative care [21]; most patients stated they need written information about palliative care and do not have the necessary information about dimensions of palliative care [22].

Lack of awareness can lead to a negative attitude towards palliative care and is an obstacle to cancer treatment. Lack of palliative care education programs for patients and their families is a barrier that reduces use of palliative care. Cancer patients need to be aware of the goals and benefits of palliative care and have access to this information through oncology of other clinical services departments [23]. The patient may be concerned about the interference of this type of care with cancer treatment [24]. Some patients think that any patient who is referred to palliative care has reached the end of his / her lifespan and death is imminent [25].Many patients consider palliative care centers as pain treatment centers [26] and misinformation has a negative effect on patients' decisions during the treatment process [27]. Unfamiliarity with palliative care can prevent the patient from being admitted to palliative care by resisting the physician’s referral to palliative care centers [28]. It is very important to identify patients’ awareness about palliative care and their decisions about participating in these services care during the remainder of their lives [29].

Palliative care is provided sporadically and in a limited number of centers in Iran, In recent years, cancer research centers and the Ministry of Health have placed an emphasis on palliative care, especially for cancer patients. Therefore, it seems that the development and progress of these programs is not far away, but there is still a long way to go to reach the desired situation, which requires collective and multi-purpose efforts [30].

Iran's health system is facing an increasing number of chronic patients, a shortage of manpower and intensive care units. Most people with incurable diseases in Iran are frequently hospitalized in the last days of life and receive specialized medication until the last moments of life, and eventually many of these patients die on hospital beds and in intensive care units [11], while in many cases, hospitalization of these patients has no effect on the patient's recovery and [12]. This increases the costs of the health system and patients' families, dissatisfaction and burnout of health professionals [14]. On the other hand, many patients prefer to spend the last days of their lives in the family and in close contact with their relatives. Evidence suggests that palliative care in Iran is provided as an island and in a limited number of centers. Most patients are deprived of this type of care and home-based palliative care in the country does not have any place in the health system [13] and patients are confused to receive palliative care and reduce the pain and suffering caused by their illness and in most cases appropriate services And is not given to these patients in a timely manner [15]. Also, the traditional view of health personnel in the treatment of incurable diseases, the lack of transparency in the law in support of health personnel to discontinue unnecessary treatment or prevent the implementation of irrational expectations of patients or their families and socio-cultural differences, are obstacles in the process of improving care. There are palliatives in Iranian society and another problem in providing palliative care is related to the obstacles in the payment process and insurance support for these patients [16, 17].

Determining the level of patients' awareness of palliative care and identifying their needs can play an important role in encouraging use of palliative care services. Developing educational resources related to palliative care for patients requires a lot of information about existing knowledge [31]. Therefore, the present study was conducted to determine the level of knowledge about palliative care in Iranian patients with cancer.

## Methods

### Ethics and consent to participate

This study is approved by Shahid Sadoughi university ethics committee with reference number *(*IR.SSU.MEDICINE.REC.1399.055*).* Informed consent from participant were obtained and the study is performed in accordance with the Declaration of Helsinki guidelines and approved by an appropriate ethics committee*.*

### Sampling method

Inclusion criteria were: having a diagnosis of cancer, 18 years and older, Persian speaking, literate, willing to participate in the study. Patients with cognitive disorders were excluded from the study. The required sample size was estimated as 94 individuals according to the study of Kolzo et al. [32], Considering the probable 10% dropout, 103 cancer patients admitted to an teaching hospital with oncology center in Yazd, Iran was selected with a convenience sample.

### Study tools

A three-part questionnaire was used to collect data: 1) Questions related to demographic information (gender, age, married, native of Iran, education, employment status, type of disease, duration of illness), 2) One question about the sources of information about palliative care, and 3) Palliative Care Knowledge Scale (PaCKS). The PaCKS was designed by Kozlov et al. (2017). It has 13 true/false questions, scored as 1 of a correct response and 0 for an incorrect response if false. Total scores range from 0–13 with higher scores indicating more knowledge. A score of 3–6 was considered a weak level of knowledge, 7–10 a moderate level, and 11–13 a good level [29].

In this study, with the permission of the instrument developer, this tool was translated from English to Persian by two independent translators simultaneously. The translators tried to translate the words within the framework of Persian culture. In the second step, the translations were compared and reconciled into a single translation. Discrepancies were identified and corrected based on a group of experts opinion. In the third step, the Persian version was given to two translators whose native language was English to be translated from Persian to English. After receiving the translations, resolving discrepancies, and merging the translations, the final translation was sent to the scale developer to verify the compatibility of the submitted version with the original version. After receiving the developer’s comments and approval of the translation, the final Persian version entered the psychometric testing process for face validity, content validity, and instrument reliability. Content validity ratio and content validity index were 0.72 and 0.79 respectively. The reliability of questionnaire was calculated using KR-20 and was 0.80, an acceptable estimate of reliability.

### Statistical analysis

The data were analyzed with using descriptive statistics of SPSS (version 21) software.

## Results

One hundred and three questionnaires were completed by the study participants. Mean age of the participants was 36.2 ± 13.5 years, 68.9% were natives of Iran, 63.1% were male, 57.3% were married, 58.3% had education at the high school and 35.9% were employed (Table 1). Type of cancer was stated as leukemia for 39.8% of participants (Table 2) and the mean duration of cancer was 2.4 ± 1.2 years. The sources of information on palliative care were: 38.8% media, 36.9% treatment team, 28.2% relatives and acquaintances, 25.2% Internet search, 19.4% books and educational pamphlets.

**Table 1:** Sample Characteristics: Demographics

Characteristics	Group	Frequency
Gender	male	65 (63.1%)
	female	38 (36.9%)
Married	no	44 (42.7%)
	yes	59 (57.3%)
Education	secondary school	11 (10.7%)
	High school	60 (58.3%)
	Associate Degree	16 (15.5%)
	Bachelor	16 (15.5%)
employment status	employee	37 (35.9%)
	worker	24 (23.3%)
	self-employment	17 (16.5%)
	unemployed	25 (24.3%)
Native	yes	71 (68.9%)
	no	32 (31.1%)

**Table 2:** Sample characteristics: Type of cancer

Type of cancer	N(%)
Osteosarcoma	7 (6.8%)
breast Cancer	9 (8.7%)
Testicles Caner	2 (1.9%)
*Prostate cancer*	2 (1.9%)
Skin cancer	3 (2.9%)
Ovarian cancer	5 (4.9%)
Lung cancer	3 (2.9%)
Liver cancer	4 (3.9%)
Colon cancer	11 (10.7%)
lymphoma	7 (6.8%)
leukemia	41 (39.8%)
Stomach cancer	1 (1%)
Neuroblastoma	3 (2.9%)
Multiple myeloma	5 (4.9%)

The mean score on the PaCKS 6.7 ± 3.7. In terms of level of awareness, the results showed that 29.1%(*n* = 30) had a good level, 50.5% (*n* = 52) had a moderate level and 20.4% (*n* = 21) had a weak level.

More than half of participants (54.1%) considered the purpose of palliative care to address psychological issues caused by an incurable disease, 64.1% stated that the stress caused by an incurable disease can be managed with palliative care, 53.3% considered palliative care to be useful in controlling the side effects of medical treatments, and 84.5% said that people should leave other doctors at the time of receiving palliative care. Many patients (71.8%) said that palliative care was only applicable to people who were in the last six months of life, 84.5% stated that palliative care is only for cancer patients, and 59.2% stated that the place of receiving palliative care is the hospital.

According to 27.2%, palliative care was designed specifically for the elderly, 70.9% considered palliative care as a team care approach, 51.4% stated that the purpose of palliative care was to help people better understand their treatment options, and 70.9% stated that palliative care encourages people to discontinue treatments aimed at treating their disease. Half of respondents said that the goal of palliative care was to improve a person's ability to participate in daily activities (50.5%) and that palliative care helps all family members to cope with incurable disease (50.9%) (Table 3).

**Table 3:** Patient Responses to Palliative Care Knowledge Scale Questions

Items	False	True	Correct response
A goal of palliative care is to address any psychological issues brought up by serious illness	47 (45.6%)	56 (54.40%)	yes
Stress from serious illness can be addressed by palliative care	37 (35.9%)	66 (64.1%)	yes
Palliative care can help people manage the side effects of their medical treatment	48 (46.6%)	55 (53.3%)	yes
When people receive palliative care, they must give up their other doctor	16 (15.5%)	87 (84.5%)	no
Palliative care is exclusively for people who are in the last six months of life	29 (28.2%)	74 (71.8%)	no
Palliative care is specifically for people with cancer	16 (15.5%)	87 (84.5%)	no
People must be in the hospital to receive palliative care	42 (40.8%)	61 (59.2%)	no
Palliative care is designed specifically for older adults	75 (72.8%)	28 (27.2%)	no
Palliative care is a team-based approach to care	30 (29.1%)	73 (70.9%)	yes
A goal of palliative care is to help people better understand their treatment options	50 (48.5%)	53 (51.4%)	yes
Palliative care encourages people to stop treatments aimed at curing their illness	30 (29.1%)	73 (70.9%)	no
A goal of palliative care is to improve a person’s ability to participate in daily activities	51 (49.5%)	52 (50.5%)	yes
Palliative care helps the whole family cope with a serious illness	49 (47.5%)	54 (52.4%)	yes

## Discussion

To our knowledge, this study is the first study to investigate the awareness of cancer patients about palliative care in Iran. This is important because identifying gaps in patients' awareness of palliative care can help with successful implementation of palliative care services and the resultant benefits to patients and families [33].

Among the participants in this study, the majority did not have a good level of knowledge about palliative care. Similarly, 81.3% of oncology patients in London [34], 63.1% of cancer patients in Japan [35], 61.1% of advanced cancer patients in the United States [36], 60.7% of patients with cancer in Ethiopia [37] and 54% of patients with advanced cancer in Singapore [38] do not know enough about palliative care or hospice care. Together, these findings are worrisome because the field of palliative care is advancing rapidly, yet little progress has been made on at the level of patient awareness about palliative care [34].

The results showed that only half of the patients are aware of the goals of palliative care. Patients consider hospice care to help alleviate patients' symptoms and the role of this type of care is to provide psychological and spiritual support for the patient and the patient's family and provide medical care for patients [39]. In Saudi Arabia, a survey of public awareness of palliative care found that most people see palliative care as improving patient quality of life, reducing physical suffering, providing patient comfort, reducing patient pain, and maintaining patient dignity [40]. A survey of public opinion in Northern Ireland found that most people see the goal of palliative care as reducing pain, providing comfort and maintaining dignity [41]. In Sweden, a similar study found that the goal of palliative care is to provide end-of-life care, pain relief, dignity, and easy death [42]. In the United States, a study of people's knowledge showed that people knew the purpose of palliative care was to help the patient's family to cope with illness, emotional social support, pain management, and other physical symptoms [43]. Overall, it seems that being aware of the goals of palliative care in various studies is the result of expanding the provision of palliative care and introducing it to patients in recent decades,

Another finding of the present study was that most participants stated that people should give up other physicians when receiving palliative care, and more than half of the patients reported that palliative care encouraged them to discontinue treatments aimed at treating their illness. In the United States, a study of knowledge and beliefs about palliative care showed that most people in general think that they should stop other treatments prescribed by physicians when starting palliative care [44].

In the present study, most patients stated that palliative care is only for people who are in the last six months of their lives and is exclusively for cancer patients. Consistent with this finding, a review study found that a common misconception about palliative care is that this type of care is for patients who have only 6 months left to live [34]. Another study reported that participants considered palliative care to be exclusive to cancer patients [45]. From the UK, a study on knowledge of palliative care of the general public found that participants thought that palliative care is for people who are dying, patients who are in the last days of their lives, or people with an incurable disease [46]. These findings indicate that the target group for palliative care is not yet well known to both the general public and oncology patients, and that awareness of who can benefit from palliative care should be a priority of palliative care center education efforts.

Regarding the place of receiving palliative care, half of the patients stated that they should go to hospitals to receive palliative care, indicating that they were not aware of the various centers that provide palliative care. Consistent with this finding, Chosich et al. reported that 39% of cancer patients knew that palliative care specialists could provide palliative care and 20.8% of the patients knew that palliative care could be provided by palliative care units, hospital-based counseling and outpatients units [47]. Hirai et al. stated that 18.6% of the patients who were aware of palliative care were unaware of the centers that provide this type of service [36] and in the study by Tebra et al., less than half of the patients knew the centers providing palliative care [48].

The consistent findings in this and prior studies show that, despite the several decades that have passed since the inception of palliative care, familiarizing patients with the options for specialists and centers providing palliative care has been neglected; this lack of awareness may be interfered with timely referral and use of palliative care.

Results showed that the media, treatment team, relatives and acquaintances, Internet search and reading books and educational pamphlets served as sources of patient information on palliative care.,. Other studies have reported the use of the media, relatives and friends, treatment team members as sources of information about palliative care [43, 49]. It is noteworthy that in the present study, the use of media was the most common, which may indicate a weakness in the role of health care providers in the field of palliative care. This finding points to inadequacies in undergraduate or postgraduate training of care team members in palliative care content. Currently in Iran, palliative care courses are not provided in the undergraduate nursing or general medical curriculum; palliative care is only mentioned, as a topic in courses related to oncology. Therefore, this weakness in professional education can prevent the adequate preparation of care team members in providing palliative care information for patients and affect patients’ willingness to use their care team members as a valuable source of information. With educational preparation in palliative care concepts followed by the efforts of care system managers, patient educators, and primary care providers to explain the important role of palliative care, health care professionals should become the first source of information for patients, providing anticipatory education prior to the time when patients will need and benefit from palliative care.

Books and educational pamphlets related to palliative care were reported as the least common source in the present study. Similarly, Taylor et al., found that less than 13% of inpatient units, 7% of outpatient units and 25% of daily chemotherapy units have written materials available on palliative care. To increase patients' awareness of palliative care services and eliminate misconceptions related to palliative care, it is imperative to prepare appropriate educational leaflets and make them available to patients in oncology departments to increase patient information and help start a discussion about the role of palliative care and its’ integration into the plan of care in a timely manner [50].

## Limitations

This study has some limitations, so the generalization of the results should be done with caution. The study participants were selected from only one university-affiliated teaching hospital, which is a center for providing services to cancer patients in Yazd. Due to the fact that at the time of sampling, Covid-19 disease was prevalent, the number of cancer patients referred to the hospital was reduced and they tried to visit less and more often went to offices or inpatient clinics, but patients with Leukemia requires repeated chemotherapy at intervals of several weeks, and more often than other cancer patients referred to the oncology ward. It seems that the frequency of leukemia patients in the present study is higher than other patients. Therefore, the frequency of leukemia in this study should not be considered as an indicator of the prevalence of this type of cancer in Iran.

So, due to the fact that all licensed palliative care centers in Iran provide palliative care in the same way based on the recommended procedure of the Ministry of Health of Iran, if the study were repeated in other Iranian centers, any variation in the results will likely be attributable to differences in professional staff preparation in palliative care or cultural differences of the population served by the center. As private centers become more common, comparing the results to the present study will provide valuable insights. This study did not evaluate the. level of knowledge of the hospital-based care team or the referring primary care providers. Assessing knowledge of palliative care among care providers would provide information on which to base professional education in support of expansion of palliative care services. This study only included oncology inpatients; palliative care for patients with non-cancer diagnoses who might benefit from palliative care needs further investigation in the Iranian context. 

## Conclusion

Overall, the findings of this study indicate misinformation and insufficient awareness of cancer patients about palliative care and highlight the importance of providing palliative care education. The development of educational programs to familiarize cancer patients with palliative care, its benefits, how it is provided, and how to access different centers for palliative care providers and members of the palliative care team can play an important role in improving patients' awareness of and knowledge about palliative care, an important obstacle to the effective use of palliative care.

## Data Availability

The datasets used and/or analyzed during the current study available from the corresponding author on reasonable request because our language is Persian and questionnaires were Persian.
